# In vitro fermentation end-products and rumen microbiome as influenced by microencapsulated phytonutrient pellets (LEDRAGON) supplementation

**DOI:** 10.1038/s41598-024-59697-x

**Published:** 2024-06-23

**Authors:** Chaichana Suriyapha, Srisan Phupaboon, Gamonmas Dagaew, Sukruthai Sommai, Maharach Matra, Rittikeard Prachumchai, Theerachai Haitook, Metha Wanapat

**Affiliations:** 1 Tropical Feed Resources Research and Development Center (TROFREC), Department of Animal Science, Faculty of Agriculture, Khon Kaen University, Khon Kaen, 40002 Thailand; 2 Division of Animal Science, Faculty of Agricultural Technology, Rajamangala University of Technology Thanyaburi, Thanyaburi, 12130 Pathum Thani Thailand

**Keywords:** Tropical fruit peel, Fruit by-product, Tropical herb, Plant secondary bioactive, Rumen microbiome, Biotechnology, Microbiology, Zoology

## Abstract

The objective of this study was to investigate the effect of microencapsulated bioactive compounds from lemongrass mixed dragon fruit peel pellet (MiEn-LEDRAGON) supplementation on fermentation characteristics, nutrient degradability, methane production, and the microbial diversity using in vitro gas production technique. The study was carried out using a completely randomized design (CRD) with five levels of MiEn-LEDRAGON supplementation at 0, 1, 2, 3, and 4% of the total dry matter (DM) substrate. Supplementation of MiEn-LEDRAGON in the diet at levels of 3 or 4% DM resulted in increased (p < 0.05) cumulative gas production at 96 hours (h) of incubation time, reaching up to 84.842 ml/ 0.5 g DM. Furthermore, supplementation with 3% MiEn-LEDRAGON resulted in higher in vitro nutrient degradability and ammonia–nitrogen concentration at 24 h of the incubation time when compared to the control group (without supplementation) by 5.401% and 11.268%, respectively (p < 0.05). Additionally, supplementation with MiEn-LEDRAGON in the diet led to an increase in the population of *Fibrobacter succinogenes* at 24 h and *Butyrivibrio fibrisolvens* at 12 h, while decreasing the population of *Ruminococcus albus*, *Ruminococcus flavefaciens*, and *Methanobacteriales* (p < 0.05). Moreover, supplementation of MiEn-LEDRAGON in the diet at levels of 2 to 4% DM resulted in a higher total volatile fatty acids (VFA) at 24 h, reaching up to 73.021 mmol/L (p < 0.05). Additionally, there was an increased proportion of propionic acid (C3) and butyric acid (C4) at 12 h (p < 0.05). Simultaneously, there was a decrease in the proportion of acetic acid (C2) and the ratio of acetic acid to propionic acid (C2:C3), along with a reduction of methane (CH_4_) production by 11.694% when comparing to the 0% and 3% MiEn-LEDRAGON supplementation (p < 0.05). In conclusion, this study suggests that supplementing MiEn-LEDRAGON at 3% of total DM substrate could be used as a feed additive rich in phytonutrients for ruminants.

## Introduction

Ruminant production plays a crucial role in the food chain, which is extremely beneficial for providing meat and milk as high-protein food by efficiently converting fibrous biomass and by-products^1^. However, the production of ruminants is accompanied by considerable emissions of enteric methane (CH_4_), resulting in environmental consequences that contribute to 30% of the methane released into the atmosphere and account for 6% of the total global anthropogenic greenhouse gas emissions^1–4^. In terms of animal performance, ruminal CH_4_ production leads to an energy loss ranging from 3 to 10% of the animal's gross energy intake^5^. Consequently, there is a need to develop sustainable mitigation approaches for CH_4_ emissions to enhance forage conversion efficiency without negative impacts on animal health and the environment.

Currently, there is growing interest in local feed additives, especially those derived from herb and fruit peels by-product available within commercial canning facilities or on farms^5,6^. Most of herbs and fruit peels are abundant in phytogenic active compounds such as condensed tannins and saponins, as well as antioxidant contents—nutritional components present in plants that contribute to animal health and have the potential to improve rumen fermentation as well as enhance ruminant productivity while also decreasing CH_4_ emissions from ruminants^5–8^. Those secondary bioactive compounds of plants can reduce ruminal CH_4_ synthesis in the rumen by reducing the number of methanogens and protozoa^7,9^. Previous studies^5,6,8,10–14^ demonstrated that the tropical herbs and fruit peels could be used to improve rumen fermentation as feed additives, which is beneficial to ruminants.

Dragon fruit (*Hylocereus undatus*) is a tropical fruit, and its peel represents a substantial by-product arising from either fruit processing or fresh consumption^15,16^. Dragon fruit peel is a non-toxic and biologically safe alternative source of plant-containing antioxidants, particularly phenolic compounds, as well as condensed tannins and saponins^11,15,17^. Previous studies^11,16,17^ demonstrated that the addition of dragon fruit peel powder or pellet could improve ruminal fermentation characteristics, enhance fermentation end-product, lower number of protozoa, and CH_4_ production.

Lemongrass (*Cymbopogon citratus*) is a tropical medicinal herb that includes the essential oil citronella, which has been confirmed to improve ruminant digestibility, rumen fermentation, ruminal microbial population, and microbial protein synthesis as well as decrease CH_4_ production^18,19^. The bioactive compound extracted from it holds therapeutic and growth-promoting potential in animals^18^. It also contributes to vitamin A synthesis and influences the proliferation and metabolism of various bacterial types, including those found in the rumen^19^.

Nano and microencapsulation techniques are employed to enhance stability, increase bio-accessibility, offer controlled release characteristics, and enhance the convenience of storage and handling for target components acting as active core materials enclosed within polymer walls or carrier materials^20^. These techniques could enhance the stability of core active ingredients, preserving them against negative environmental conditions including high temperatures, exposure to light, changes in pH, and the presence of oxygen, which may greatly affect the chemical and physical properties of the product^15,21^. Phupaboon et al.^20^ achieved microencapsulation to encapsulate high-quality bioactive substances extracted from *Mitragyna speiosa*, *Cannabis indica*, and *Cannabis sativa*, which was effective in preserving elevated concentrations of bioactive components. Recently, Matra et al.^22^ demonstrated that microencapsulated phytonutrients from *Mitragyna* leaf extracts have the potential to improve ruminal degradability, enhance fermentation end-products, and decrease methane production in an in vitro study. It is a new challenge to apply the microencapsulation process to increase the efficiency of the use of bioactive compounds in the feed industry.

However, research on microencapsulated-phytonutrients from lemongrass mixed dragon fruit peel pellet (MiEn-LEDRAGON) has not been reported. The study’s hypothesis was that MiEn-LEDRAGON could be effectively retained as active in the in vitro inoculum and improve fermentation characteristics, degradability, and microbial diversity, as well as mitigate CH_4_ production. Therefore, the objective of this study was to determine the effect of MiEn-LEDRAGON supplementation on fermentation characteristics, degradability, methane production, and microbial diversity using in vitro gas production technique.

## Materials and methods

All cattle donors involved in this study received approval from the Animal Ethics Committee of Khon Kaen University (record no. IACUC-KKU-110/66). Our study confirmed that all the methodologies employed in this research adhered to the pertinent guidelines and regulations. Moreover, strict adherence to the ARRIVE guidelines was maintained throughout the entire study.

### Microencapsulation of phytonutrients product pellet preparation

The phytonutrients product pellet from lemongrass mixed dragon fruit peel (LEDRAGON) – contains lemon grass powder (50%), dragon fruit peel powder (45%), molasses (3%), and cassava powder (2%) on a dry matter basis. All feed ingredients were meticulously combined and mixed with water, then processed through a pelleting machine to form the LEDRAGON pellets. Subsequently, the pellets were sun-dried for 48 h to achieve a moisture level of at less than 10%. These pellets were stored in a sealed container and utilized throughout the entire experimental duration. Subsequently, the LEDRAGON pellet was ground to powder for plant secondary bioactive substance extraction, modifying the procedure outlined by Phupaboon et al.^20^. Specifically, 5 g of LEDRAGON pellet powder was mixed with 100 mL of deionized water and subjected to microwave extraction at 100 W for 10 min (final temperature ≤ 60 °C)^20^. The resulting bioactive extract juice was encapsulated using a technique involving ionic gelation in combination with surfactant ingredients, utilizing 10% cricket-extracted protein (w/v) in phosphate buffer (PPB) at pH 5.5. The bioactive extract juice was stirred overnight at room temperature with surfactant ingredients in a 1:1 ratio. The bioactive substance was microencapsulated using the spray-drying technique with a Bǚchi B-191 mini spray dryer. The dried microencapsulated-LEDRAGON powders (MiEn-LEDRAGON) were gathered, tightly packed, sealed, and kept at a temperature of − 20 °C until used in the in vitro investigation.

### Experimental design and dietary treatments

This study utilized the gas production technique at various incubation intervals. The design of experiment was a completely randomized design (CRD) with three replications run. The dietary treatments were basal diet (rice straw + concentrate diet) with a 60:40 of roughage to concentrate (R:C) ratio supplemented with MiEn-LEDRAGON at 0, 1, 2, 3, and 4% DM of total substrate. The experimental dietary samples were dried in an oven at 72°C, ground to pass through a 1-mm sieve using a Cyclotech Mill (Tecator, Sweden), and then analyzed for chemical composition as well as in the in vitro gas production test. The experimental diets, comprising rice straw, concentrate diet, dragon fruit peel, lemon grass, pellet product from lemongrass mixed dragon fruit peel (LEDRAGON), and MiEn-LEDRAGON, underwent analysis for dry matter (DM, ID 967.03) and ash (ID 492.05) content, employing the standard procedures of AOAC^23^. The amount of nitrogen (N) components was determined using the Nitrogen Analyzer (Leco FP828, LECO Corporation, Saint Joseph, MI, USA) to evaluate the crude protein (CP) content. The content of neutral detergent fiber (NDF) and acid detergent fiber (ADF) was determined following the standard method outlined by Van Soest et al.^24^. Total phenolic content was assessed using the Folin–Ciocalteu reagent through absorbance measurement at 765 nm^25^. The total flavonoid content was evaluated by measuring colorimetric changes using a 10% aluminium chloride solution and reading at 415 nm^26^. The analyses were conducted in triplicate, and the outcomes were presented as mg of gallic acid equivalents (mg GAE) per g of DM and mg of quercetin equivalents (mg QUE/g DM), following the description of Phupaboon et al.^20^ The assessment of antioxidative capacities involved three distinct methods: 2, 2-diphenyl-1-picrylhydrazyl (DPPH) radical scavenging method^27^, 2, 2′-azino-bis (3-ethylbenzthiazoline-6-sulphonic acid) (ABTS) radical scavenging activity^28^, and ferric reducing ability power (FRAP) method^29^. The analyses were conducted in triplicate, and the outcomes were presented as the % of radical scavenging inhibition and mmol of Trolox equivalents (mmol TROE/g DM), following the description of Phupaboon et al.^20^. The compositions and ingredients of the experimental diets, comprising rice straw, concentrate diet, dragon fruit peel, lemon grass, LEDRAGON, and MiEn-LEDRAGON are shown in Table 1. All concentrate diets being formulated to be at 14.6% DM of CP, which was recommend for beef cattle.

**Table 1:** Feed ingredients and chemical composition of concentrate diet, rice straw, dragon fruit peel, lemon grass, pellet product from lemongrass mixed dragon fruit peel (LEDRAGON), and microencapsulation of LEDRAGON used in the experiment.

Item	Concentrate diet	Rice straw	Dragon fruit peel	Lemongrass	LEDRAGON^5^	MiEn-LEDRAGON^6^
Ingredient, g/kg of dry matter						
Cassava chip	540	–	–	–	–	–
Rice bran	170	–	–	–	–	–
Palm kernel meal	130	–	–	–	–	–
Soybean meal	105	–	–	–	–	–
Urea	25	–	–	–	–	–
Sulphur	10	–	–	–	–	–
Salt	10	–	–	–	–	–
Mineral premix^1^	10	–	–	–	–	–
Chemical compositions						
Dry matter (DM), g/kg	905	901	935	692	913	922
Organic matter, g/kg of DM	922	854	963	901	921	988
Crude protein, g/kg of DM	146	24	52	41	62	264
Neutral detergent fiber, g/kg of DM	204	789	371	663	412	602
Acid detergent fiber, g/kg of DM	126	526	289	403	294	201
Total phenolic contents, mg GAE/g DM	–	–	851	386	585	1254
Total flavonoid contents, mg QUE/g DM	–	–	63	7	24	264
DPPH^2^, mg TROE/g DM	–	–	2,982,500	1,963,611	3,355,833	2,430,278
ABTS^3^, mg TROE/g DM	–	–	1,472,500	1,664,722	1,335,833	1,756,944
FRAP^4^, mg TROE/g DM	–	–	23,558	7492	14,869	13,192

^1^Minerals and vitamins (each kg contains): vitamin A: 10,000,000 IU, vitamin E: 70,000 IU, vitamin D: 1,600,000 IU, Fe: 50 g, Zn: 40 g, Mn: 40 g, Co: 0.1 g, Cu: 10 g, Se: 0.1 g, I: 0.5 g, ^2^DPPH = 2,2-diphenyl-1-picrylhydrazyl, ^3^ABTS = 2,2′-azino-bis (3-ethylbenzthiazoline-6-sulphonic acid), ^*4*^*FRAP* ferric reducing ability power, ^5^*LEDRAGON* lemongrass mixed dragon fruit peel, ^6^*MiEn*-*LEDRAGON* microencapsulation of LEDRAGON.

### Animal donors and preparation of ruminal inoculums

Four, male 4-year-old, Thai-native steers with 380 ± 10.0 kg of body weight (BW) were used as ruminal liquor donors. The experimental donor cattle were given ad libitum access to rice straw and provided with 0.5% of their body weight (BW) daily for the concentrate diet (145 g/kg CP and 810 g/kg TDN), twice a day (6:00 am and 4:00 pm). All experimental donors were housed in individual pens, and free available was made for clean water and mineral blocks. The cattle were provided with the diet for a period of 21 days before the collection of rumen liquor. For each incubation run, ruminal fluid was collected from each donor animal for 300 mL via oral suction using a vacuum pump and then transferred to an Erlenmeyer flask before the morning feeding. Ruminal fluid was filter strained through four layers of cheesecloth into pre-warmed thermos containers before being transferred to the laboratory. The medium preparation adhered to the procedures outlined by Makkar et al.^30^, where 2400 mL of ruminal buffer mixed medium was combined with 1200 mL of ruminal fluid obtained from animal donors and stirred at 39 °C under CO_2_-flushed conditions. Ruminal fluid mixture (40 mL) was moved into each experimental bottle and incubated at 39 °C in a water bath.

### In vitro gas production and kinetics of gas

Gas productions were promptly recorded using the modified procedures described by Suriyapha et al.^31^ immediately after incubation at 0, 0.5, 1, 2, 4, 6, 8, 12, 18, 24, 48, 72, and 96 h. The gas kinetics were analyzed by fitting curves using the models proposed by Ørskov and McDonald^32^ as follows:

$${\text{y}}\, = \,{\text{a}}\, + \,{\text{b }}({1}{-}{\text{e}}^{{({-}{\text{ct}})}} ).$$

where a = soluble fraction from gas production, b = insoluble fraction from gas production, c = rate of gas production constant for the insoluble fraction (b), t = incubate time, (|a|+ b) = the potential extent of gas production, and y = gas produced at time ‘t’.

### In vitro degradability, fermentation characteristics and microbial DNA analysis

The analysis of in vitro dry matter degradability (IVDMD) and in vitro organic matter degradability (IVOMD) was conducted following 12 and 24 h of incubation, in accordance with the methods outlined by Tilley and Terry^33^. At 12 and 24 h, the gas that was in the empty area in the headspace of the bottle was retrieved using a 10 mL syringe and transferred into the vial to collect methane. The concentration of methane was assessed utilizing high-purity methane as a reference standard under the gas chromatography (GC) system (Nexis GC-2030: SHIMADZU, Shimadzu Corp., Kyoto, Japan) equipped with a standard column (SH-Rt-Q-BOND 30 m, 0.53 mm, 20 μm, Shimadzu Co., Tokyo, Japan) following the description of Kaewpila et al.^34^. The ruminal pH parameters were measured at 12 and 24 h of incubation using a digital pH meter (HANNA Instrument (HI) 8424 microcomputer, Singapore). The ruminal liquor incubated bottles were distributed into two parts. The first part (20 mL) was kept into 5 mL of 1 M H_2_SO_4_ and stored at – 20 °C for ammonia nitrogen (NH_3_–N) analysis using a spectrophotometer (UV/VIS spectrophotometer, PG Instruments LtD., London, United Kingdom) based on the methods of Fawcett and Scott^35^, while the measurement of in vitro volatile fatty acids (VFAs) was conducted using the GC system (Nexis GC-2030), which included the molecular sieve 13X, 30/60 mesh column (Alltech Associates Inc., Deerfield, IL, USA), following the description of So et al.^36^. The second part (10 mL) was collected into a plastic bottle and stored at – 20 °C for microbial extraction. The procedure described by Koike and Kobayashi^37^ was utilized for the extraction of community DNA from rumen fluid. DNA purification was performed using the QIAgen DNA Mini Stool Kit columns (QIAGEN, Valencia, CA, USA). Specific primers, as described by Matra et al.^22^, were applied to quantify the microbial populations of *Fibrobacter succinogenes*, *Ruminococcus albus* and *Ruminococcus flavefaciens*^37^, *Butyrivibrio fibrisolvens*^38^, *Megasphaera elsdenii*^39^, and *Methanobacteriales*^40^. The Chromo 4TM system from Bio-Rad (Hercules, CA, USA) was employed for real-time PCR amplification and detection, following the guidelines for DNA analysis^37^.

### Statistical analysis

The investigation results were statistically analyzed as a completed randomized design (CRD) statistical run with Proc. Mixed procedure in SAS software^41^ (Version 9.4):

$${\text{Y}}_{{{\text{ij}}}} = \, \mu \, + \, \tau_{{\text{i}}} + \, \delta_{{{\text{ij}}}} + \, \varepsilon_{{{\text{ijk}}}} ,$$

where Y_ij_ = each observations for a given variable, µ = overall mean, τ_i_ = the fixed effect of treatment (the MiEn-LEDRAGON levels at 0, 1, 2, 3, and 4% DM of total substrate), δ_ij_ = the random effect of the replication run, and ε_ijk_ = the residual random term. Results are presented as mean values with the standard error of the mean. Differences between treatment means were determined by the Duncan’s New Multiple Range Test^42^, and differences of p < 0.05 were considered to represent statistically significant differences.

## Result

### Kinetics and cumulative gas production

**Figure 1:**
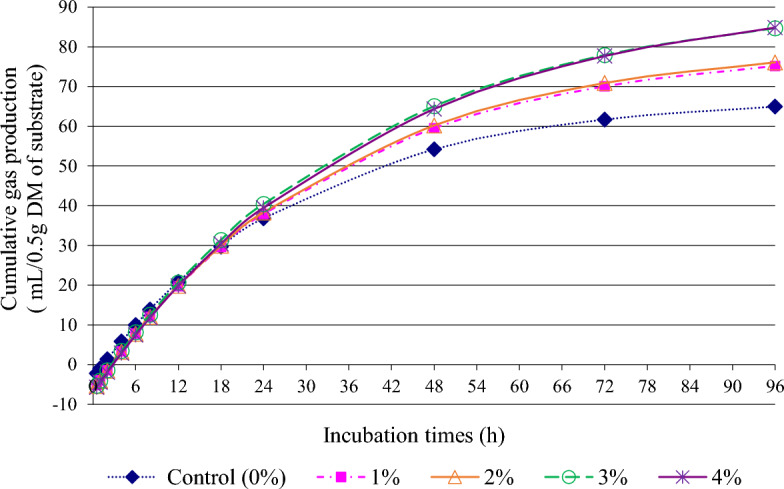
Effect of microencapsulated phytonutrients from lemongrass mixed dragon fruit peel pellet (MiEn-LEDRAGON) supplementation levels on in vitro cumulative gas production during incubation times.

The effect of MiEn-LEDRAGON supplementation levels on gas kinetics and cumulative gas production is shown in Table 2. The soluble fraction of gas production (a) was decreased (p < 0.05) when supplemented with MiEn-LEDRAGON (1 to 4% DM in the diet). The insoluble fraction of gas production (b) value increased significantly (p < 0.05) upon supplementation with MiEn-LEDRAGON, reaching its highest value at the 3 or 4% MiEn-LEDRAGON supplementation level. The rate of gas production value (c) exhibited a decrease upon supplementation with MiEn-LEDRAGON in the diet (p < 0.05). Furthermore, the potential extent of gas (|a|+ b) value and cumulative gas production were increased with MiEn-LEDRAGON supplementation at 3 or 4% DM in the diet (p < 0.05), and cumulative gas production ranged from 64.95 to 84.84 mL/0.5g DM of substrate (Fig. 1).

**Table 2:** Effect of microencapsulated phytonutrients from lemongrass mixed dragon fruit peel pellet (MiEn-LEDRAGON) supplementation levels on gas kinetics and cumulative gas at 96 h after incubation.

Items	MiEn-LEDRAGON levels					SEM^2^	*p*-value
	0%	1%	2%	3%	4%		
Gas kinetics^3^							
a	− 3.410^a^	− 6.430^b^	− 6.692^b^	− 6.618^b^	− 6.990^b^	0.382	< 0.001
b	70.821^c^	86.479^b^	87.701^b^	98.710^a^	100.080^a^	0.481	< 0.001
c	0.035^a^	0.030^ab^	0.030^ab^	0.027^b^	0.026^b^	0.002	0.001
|a|+ b	74.231^c^	92.909^b^	94.393^b^	105.328^a^	107.070^a^	0.579	< 0.001
Cumulative gas, mL/0.5g DM	64.950^c^	75.195^b^	76.087^b^	84.700^a^	84.842^a^	1.333	< 0.001

^a–c^Value on the same row with different superscripts differ (p < 0.05), ^1^*MiEn*-*LEDRAGON* microencapsulation of pellet product from lemongrass mixed dragon fruit peel (LEDRAGON), ^2^*SEM* standard error of the mean, ^3^a = the gas production from the immediately soluble fraction, b = the gas production from the insoluble fraction, c = the gas production rate constant for the insoluble fraction (b), |a|+ b = the gas potential extent of gas production.

### In vitro degradability, pH value and ammonia–nitrogen concentration

Table 3 shows the effect of MiEn-LEDRAGON supplementation levels on in vitro dry degradability, pH value, and concentration of ammonia–nitrogen (NH_3_–N). There were no changes in IVDMD, IVOMD, and NH_3_–N at 12 h of incubation time (p > 0.05). Whereas, the greater IVDMD, IVOMD, and NH_3_–N at 24 h of incubation time were shown in MiEn-LEDRAGON supplementation at 3 or 4% DM in the diet (p < 0.05). However, the pH value did not change (p > 0.05) when MiEn-LEDRAGON was supplemented in the diet.

**Table 3:** Effect of microencapsulated phytonutrients from lemongrass mixed dragon fruit peel pellet (MiEn-LEDRAGON) supplementation levels on in vitro degradability, in vitro pH and ammonia–nitrogen concentration.

Items	MiEn-LEDRAGON^1^ levels					SEM^2^	*p*-value
	0%	1%	2%	3%	4%		
In vitro dry matter degradability, g/kg							
12 h	492	492	490	491	490	3.924	0.91
24 h	537^b^	543^b^	550^b^	566^a^	563^a^	4.609	0.002
In vitro organic matter degradability, g/kg DM							
12 h	493	493	492	493	492	3.833	0.904
24 h	562^b^	569^b^	572^b^	586^a^	585^a^	4.269	0.002
pH value							
12 h	6.860	6.850	6.860	6.850	6.850	0.006	0.979
24 h	6.933	6.909	6.920	6.900	6.900	0.009	0.941
Ammonia–nitrogen, mg/dL							
12 h	8.281	8.297	8.273	8.299	8.309	0.072	0.943
24 h	10.002^b^	10.301^b^	10.309^b^	11.053^a^	11.129^a^	0.183	0.011

^a,b^Value on the same row with different superscripts differ (p < 0.05), ^1^*MiEn-LEDRAGON* microencapsulation of pellet product from lemongrass mixed dragon fruit peel (LEDRAGON), ^2^*SEM* standard error of the mean.

### In vitro microbial dynamics

The effect of MiEn-LEDRAGON supplementation levels on in vitro microbial populations is shown in Table 4. The supplementation of MiEn-LEDRAGON had no impact on the *Fibrobacter succinogenes* population at 12 h of incubation (p > 0.05), whereas a greater number of *Fibrobacter succinogenes* were observed at 24 h of supplementation with MiEn-LEDRAGON in the diet at levels of 3 or 4% DM (p < 0.05). In addition, the number of *Butyrivibrio fibrisolvens* at 12 h of incubation was higher (p < 0.05) when MiEn-LEDRAGON was added to the diet at 3 or 4% DM, nevertheless, there was no change for the *Butyrivibrio fibrisolvens* population at 24 h (p > 0.05). Meanwhile, the populations of *Ruminococcus albus* and *Ruminococcus flavefaciens* at 12 h and 24 h were decreased (p < 0.05) when supplemented with MiEn-LEDRAGON (1 to 4% DM in the diet). Moreover, the supplementation of MiEn-LEDRAGON at 2 to 4% DM showed a lower *Methanobacteriales* population at 12 h and 24 h (p < 0.05). However, there was no change for the *Megasphaera elsdenii* population by dietary treatment (p > 0.05).

**Table 4:** Effect of microencapsulated phytonutrients from lemongrass mixed dragon fruit peel pellet (MiEn-LEDRAGON) supplementation levels on in vitro microbial population.

Items	MiEn-LEDRAGON^1^ levels					SEM^2^	*p*-value
	0%	1%	2%	3%	4%		
Log_10_ copies/mL of rumen content							
* Fibrobacter succinogenes*							
12 h	9.233	9.233	9.236	9.235	9.234	0.024	0.959
24 h	9.718^c^	9.718^c^	9.791^b^	9.863^a^	9.869^a^	0.019	0.011
* Ruminococcus albus*							
12 h	9.202^a^	9.148^ab^	9.124^ab^	9.103^b^	9.091^b^	0.028	0.021
24 h	9.834^a^	9.770^ab^	9.693^ab^	9.661^b^	9.660^b^	0.037	0.011
* Ruminococcus flavefaciens*							
12 h	9.268^a^	9.242^ab^	9.213^ab^	9.188^b^	9.190^b^	0.020	0.020
24 h	9.529^a^	9.497^ab^	9.468^b^	9.447^b^	9.448^b^	0.018	0.011
* Butyrivibrio fibrisolvens*							
12 h	7.491^c^	7.936^b^	7.862^b^	8.020^ab^	8.271^a^	0.086	0.001
24 h	8.517	8.514	8.516	8.520	8.519	0.078	0.914
* Megasphaera elsdenii*							
12 h	10.239	10.233	10.149	10.160	10.271	0.034	0.628
24 h	10.518	10.520	10.488	10.501	10.511	0.032	0.761
* Methanobacteriales*							
12 h	8.311^a^	8.162^a^	7.711^b^	7.717^b^	7.803^b^	0.065	0.001
24 h	8.511^a^	8.411^a^	8.333^ab^	8.148^b^	8.148^b^	0.066	0.001

^a–c^Value on the same row with different superscripts differ (p < 0.05), ^1^*MiEn-LEDRAGON* microencapsulation of pellet product from lemongrass mixed dragon fruit peel (LEDRAGON), ^2^*SEM* standard error of the mean.

### In vitro volatile fatty acids and methane production

Table 5 shows the effect of MiEn-LEDRAGON supplementation levels on in vitro fermentation end-products (VFA) and production of methane (CH_4_). Even though the total VFA at 12 h was similar across all dietary groups (p > 0.05), the supplementation of MiEn-LEDRAGON in the concentrate diet at levels of 2 to 4% DM resulted in a greater total VFA (p < 0.05) at 24 h after incubation than the control group. The proportion of acetic acid (C2) at 12 h and 24 h were decreased (p < 0.05) with the MiEn-LEDRAGON supplemented levels of 2 to 4% DM in the diet. Meanwhile, there was an increased proportion of propionic acid (C3) (p < 0.05) when supplemented with MiEn-LEDRAGON in the diet at levels of 2 to 4% DM in the diet. Furthermore, the supplementation of MiEn-LEDRAGON in the concentrate diet showed a higher proportion of butyric acid (C4) at 12 h (p < 0.05) when compared with the control group, whereas there was no change (p > 0.05) in the C4 proportion at 24 h. The ratio of acetic acid to propionic acid (C2:C3) at 12 h and 24 h were decreased (p < 0.05) with the MiEn-LEDRAGON supplemented levels of 2 to 4% DM in the diet. Furthermore, the in vitro CH_4_ production at 12 h decreased (p < 0.05) with the supplementation of MiEn-LEDRAGON (1 to 4% DM) in the diet. Additionally, there was an observed lower CH_4_ production at 24 h with the MiEn-LEDRAGON supplementation levels ranging from 2 to 4% DM in the diet (p < 0.05).

**Table 5:** Effect of microencapsulated phytonutrients from lemongrass mixed dragon fruit peel pellet (MiEn-LEDRAGON) supplementation levels on in vitro volatile fatty acids and methane production.

Items	MiEn-LEDRAGON^1^ levels					SEM^2^	*p*-value
	0%	1%	2%	3%	4%		
Total volatile fatty acids, mmol/L							
12 h	52.133	53.112	53.231	52.260	52.151	0.413	0.922
24 h	69.321^b^	69.263^b^	71.343^a^	73.021^a^	70.980^ab^	0.540	0.010
Acetic acid, mol/100 mol							
12 h	58.660^a^	58.701^a^	57.440^ab^	56.280^b^	56.132^b^	0.489	0.003
24 h	60.429^a^	59.691^a^	57.519^b^	57.930^b^	57.610^b^	0.499	0.004
Propionic acid, mol/100 mol							
12 h	29.989^b^	29.609^b^	30.709^ab^	31.601^a^	31.709^a^	0.339	0.001
24 h	29.240^b^	30.029^b^	32.059^a^	31.758^a^	32.080^a^	0.430	0.001
Butyric acid, mol/100 mol							
12 h	11.351^c^	11.690^b^	11.851^b^	12.119^a^	12.159^a^	0.069	0.001
24 h	10.331	10.280	10.422	10.312	10.410	0.101	0.912
Acetic acid to propionic acid ratio							
12 h	1.956^a^	1.983^a^	1.870^ab^	1.781^b^	1.770^b^	0.037	0.001
24 h	2.067^a^	1.988^a^	1.794^b^	1.824^b^	1.796^b^	0.038	0.001
Methane production, mL/100 mL of total gas							
12 h	2.423^a^	2.312^ab^	2.189^b^	2.159^b^	2.213^b^	0.057	0.001
24 h	6.422^a^	6.310^a^	5.823^b^	5.671^b^	5.710^b^	0.109	0.001

^a–e^Value on the same row with different superscripts differ (p < 0.05), ^1^*MiEn*-*LEDRAGON* microencapsulation of pellet product from lemongrass mixed dragon fruit peel (LEDRAGON), ^2^*SEM* standard error of the mean.

## Discussion

In this study, the gas production from the immediately soluble fraction (a) was shown to have a negative value. Several studies^31,43–46^ indicate that when mathematical models were used to match with gas output kinetics, negative values were recorded for a variety of substrates and microbial activity. This occurred due to the delayed establishment of ruminal microbe growth on the substrates in the initial phase of incubation^31,43,45^. This situation indicates the presence of a gap time and delay period after the ingestion of the soluble portion of the substrate, prior to the fermentation of the cell walls^44–46^. Therefore, the application of the absolute value of 'a' (|a|) has been recognized as a means to describe the optimum fermentation of the soluble fraction^31,44,45^. In this study, higher gas production of (|a|) was observed in the MiEn-LEDRAGON supplementation group (1 to 4% DM). This increase could be attributed to the elevated levels of oligosaccharides in dragon fruit peel, particularly fructooligosaccharides (FOS), known as a soluble dietary fraction^47–49^, which was extracted in MiEn-LEDRAGON. The soluble fraction facilitates binding with rumen microbes, contributing to a higher production of gas^31,43,46^.

The present study observed an increased gas production of (b) with rising MiEn-LEDRAGON levels, possibly attributed to the protein-based polymers constituting the polymer walls or carrier materials of MiEn-LEDRAGON. Generally, proteins are one type of carrier material for microencapsulation that are in their native state within the environment, as they are typically insoluble or bound without undergoing degradation in most instances, but it is soluble in diluted acids or bases in minute quantities^50^. Therefore, the elevated insoluble content could be a contributing factor to the higher gas production of (b). In a recent study, Matra et al.^22^ demonstrated that the supplementation of microencapsulated *Mitragyna* leaves extract at 4 to 6% DM in the diet led to increased gas (b) in an in vitro study.

Typically, a higher soluble fraction is associated with a rapid gas production rate (c)^31,44,46,51^. However, contrary to this expectation, our study found a lower rate of gas production (c) in the MiEn-LEDRAGON supplemented group. This observation may be attributed to the increased presence of the insoluble fraction derived from the polymer wall materials of MiEn-LEDRAGON. Chumpawadee et al.^45^ revealed that a higher insoluble fraction in feed affects slow microbial degradation. The breakdown of the insoluble fraction occurred at a rate approximately four times slower than the breakdown of the soluble fraction during anaerobic digestion^52^. This leads to a decline in the microbial degradation efficiency in the rumen, causing a gradual reduction or slowdown in the degradation process, which is to achieve higher and longer stability of the core substrate within the ruminal inoculum^53,54^. Similarly, Ibrahim and Hassen^55^ demonstrated that the slow release provided by microencapsulated tannin effectively protects the bioactive compound, leading to a slow degradation rate until it reaches the target site.

In this study, a greater potential extent of gas production (|a|+ b) for the MiEn-LEDRAGON supplemented group could be due to a higher mathematical result from the sum of (|a|) and (b), which is primarily the consequence of the carbohydrate fermentation into VFA^31,51^. The present study, the observed increase in production of cumulative gas at 96 h after incubation with rising MiEn-LEDRAGON levels (3 or 4%) may be attributed to the phytonutrient content of MiEn-LEDRAGON. This includes total phenolic and flavonoid contents, as well as antioxidative substances, which likely have a beneficial ability to interact with fiber and protein contents, leading to enhanced ruminal fermentation and gas production^6,16,22,56^. Matra et al.^16^ suggested that the supplementation of dragon fruit peel at 4% of total substrate could increase the gas production and ruminal fermentation. Similarly, Matra et al.^22^ revealed that the use of microencapsulated phytonutrients from *Mitragyna* leaf extracts could enhance gas production and ruminal fermentation.

In this study, the similarity in both in vitro degradability of IVDMD and IVOMD at 12 h after incubation could be attributed to the deceleration and increased stability of the core substrate facilitated by the microencapsulated wall of MiEn-LEDRAGON in the in vitro degradation process^53,54^. This effect may lead to a temporary slowdown in the efficiency of microbial decomposition, resulting in similar outcomes for a short period^55^. Consequently, this might lead to similarities in cumulative gas production for the first 12 h and in vitro degradability at 12 h. While the present study revealed a greater in vitro degradability (IVDMD and IVOMD) at 24 h after incubation for added MiEn-LEDRAGON at 4 or 6% DM of total substrate. This might result from the phytonutrients, particularly flavonoids and phenolics, present in MiEn-LEDRAGON, which exhibit various biological effects that can influence ruminal microbes, ultimately enhancing the degradation of feed in the rumen^22,57,58^. In addition, this might be a result of the balanced concentrations of condensed tannins and saponin derived from the dragon fruit peel in MiEn-LEDRAGON, influencing rumen fermentation^22,59^. Moreover, it could be attributed to the vital nutrients promoting microbial activity sourced from the dragon fruit peel in MiEn-LEDRAGON, potentially enhancing the degradability of nutrients. Matra et al.^16^ demonstrated that the supplementation of dragon fruit peel at 4% of total substrate could enhance the in vitro degradability. Similarly, Matra et al.^22^ indicated that microencapsulated phytonutrients from *Mitragyna* leaf extracts could promote the in vitro degradability.

The present study indicates that the dietary treatments did not affect rumen pH within the normal range of 6.85 to 6.93. Wanapat et al.^60^ revealed that pH levels between 6.5 and 7.0 are considered optimal for ruminal fermentation, microbial activity, and microbial growth. Furthermore, the previous studies^8,61^ revealed that the incorporation of feed ingredients rich in phenolic compounds can improve rumen fermentation by maintaining the ruminal pH.

In this study, NH_3_–N concentration at 24 h after incubation was enhanced with MiEn-LEDRAGON supplementation at 4 or 6% DM. This may be attributed to the high CP level in MiEn-LEDRAGON (264 g/kg of CP), which is formulated with a cricket protein extract as a polymer wall carrier material. Furthermore, this is probably due to the capability of the plant bioactive extract to augment the proteolysis process^22^. According to Ahmed et al.^62^, the addition of plant-derived bioactives leads to an elevation in ruminal NH_3_–N concentration. Similarly, Matra et al.^22^ demonstrated that the microencapsulated phytonutrients from *Mitragyna* leaf extracts supplementation could enhance in vitro NH_3_–N concentration.

This study's results demonstrated a lower population of both cellulolytic *Ruminococcus* species (*R. albus* and *R. flavefaciens*) when MiEn-LEDRAGON was added to the diet at 3 or 4% DM. This could be attributed to the susceptibility of bio-active compounds namely phenolic and flavonoid compounds rich in MiEn-LEDRAGON^1,63^. This finding aligns with Kim et al.^63^, who reported a decrease in the population of *R. albus* and *R. flavefaciens* when flavonoid plant extract was included in the diet compared to the control. Similarly, several previous studies^1,64,65^ demonstrated that the presence of plant secondary compounds such as flavonoid, phlorotannin, and condensed tannin resulted in a reduction in the abundance of *R. albus* and *R. flavefaciens* compared to the control. Moreover, this study interestingly showed an enhancement in the population of another major cellulolytic bacterium, *F. succinogenes*, at 24 h after incubation with MiEn-LEDRAGON supplementation at 3 or 4% DM. Given that phenolic and flavonoids typically exert antimicrobial effects by inhibiting cytoplasmic membrane function, hindering bacterial cell wall synthesis, or impeding nucleic acid synthesis^66,67^, differences in sensitivity to these plant secondary compounds may arise due to variations in the cell wall structures of Gram-negative bacteria (*F. succinogenes*) and Gram-positive bacteria (*Ruminococcus* species)^1,9^. Therefore, after a reduction in the populations of the two cellulolytic *Ruminococcus* species, there might be a compensatory rise in *F. succinogenes* populations. Kim et al.^63^ confirmed that an increase in *F. succinogenes* diversity when exposed to flavonoid plant extract, whereas populations of *R. albus* and *R. flavefaciens* decreased compared to the control. Similarly, Chen et al.^1^ revealed that the population of *F. succinogenes* increased with tannin treatments, while the populations of *R. albus* and *R. flavefaciens* decreased. This study's findings indicate that supplementing MiEn-LEDRAGON increased the population of *Butyrivibrio fibrisolvens* at 12 h. This enhancement might be attributed to MiEn-LEDRAGON promoting the breakdown of cellulose and protein by boosting the populations of bacteria involved in decomposing these plant bioactive components^58,67^. Matra et al.^22^ demonstrated a similar outcome with the addition of microencapsulated bioactive compounds from *Mitragyna* leaf extracts at 6% DM, which stimulated the *B. fibrisolvens* population. Additionally, Zhan et al.^58^ showed that alfalfa flavonoids also contribute to the growth of *B. fibrisolvens* in dairy cows. Meanwhile, this study indicates a decrease in the methanogen group (*Methanobacteriales*) when MiEn-LEDRAGON was added to the diet, which could be attributed to the presence of bioactive compounds rich in MiEn-LEDRAGON. Plant secondary compounds promptly influences rumen methanogens by interacting with the proteinaceous adhesin, suppressing methanogen growth, decreasing interspecies carbon and hydrogen transfer, and preventing the formation of the methanogen-protozoa complex^1,68,69^. Patra et al.^70^ found that extracts containing phenolics led to a reduction in ruminal methane emission and the number of protozoa. Similarly, Patra and Saxena^9^ demonstrated that flavonoids exert direct effects against methanogens, leading to a decrease in protozoa associated with ruminal methanogenesis. Matra et al.^22^ reported that the supplementation of microencapsulated bioactive compounds from *Mitragyna* leaf extracts has potential to decrease the *Methanobacteriales* population.

In this study, a higher total VFA at 24 h after incubation was observed in the MiEn-LEDRAGON supplementation groups (at 2 to 4% DM). This increase could be attributed to enhanced degradability. Furthermore, the plant secondary compounds in MiEn-LEDRAGON likely played a significant role in supporting microbial fermentation, contributing to the increase and benefiting the production of ruminal fermentation end-products (VFA)^5,10,12^. Matra and Wanapat^11^ revealed that the phytonutrients from dragon fruit peel pellets promoted total VFA production. Particularly, MiEn-LEDRAGON supplementation groups (at 2 to 4% DM) exhibited a higher proportion of C3, while a lower proportion of C2, C2:C3 ratio, and CH_4_ production. This may be attributed to the potential of plant secondary compounds to influence the production of C3 in conditions of hydrogen excess^10^. In such situations, hydrogen may be utilized to generate propionate instead of serving as the primary substrate for methane production^71^. Furthermore, the increased C3 production due to MiEn-LEDRAGON supplementation might be associated with the elevated population of *F. succinogenes*, known as a succinate-producing microbe in the rumen^1^. This heightened C3 production can be attributed to the succinate–propionate pathway, a predominant route for the C3 production in the rumen^72^. Additionally, this probably due to a consequence of the reduction in the population of *R. albus* and *R. flavefaciens*, which are recognized as substantial hydrogen (H_2_) producers and have been shown to produce CH_4_ when co-cultured with methanogens^1,64^. On the contrary, the increased abundance of *F. succinogenes*, a non-hydrogen producer, contributes to elevated C3 production, a decreased C2:C3 ratio, and a reduction in CH_4_ production^1,73^. Matra and Wanapat^11^ demonstrated that phytonutrients from dragon fruit peel pellets increased the proportion of C3, while decreasing the proportion of C2 and CH_4_ production. Similarly, Matra et al.^22^ reported that the supplementation of microencapsulated bioactive compounds from *Mitragyna* leaf extracts has the potential to enhance C3 production and reduce CH_4_ production. This study, a higher C4 proportion at 12 h could be due to an increasing of *B. fibrisolvens*, a major butyrate-producing bacterium in the rumen at 12 h after incubation^74^. Similarly, Matra et al.^22^ revealed that the addition of microencapsulated bioactive compounds from *Mitragyna* leaf extracts in the diet was increased C4 proportion.

Inconclusion, this study suggests that supplementing MiEn-LEDRAGON at 3% of total DM substrate enhances cumulative gas production, in vitro degradability, and NH_3_-N concentration as well as improved microbial diversity and fermentation end-products, particularly C3 production, while decreasing *Methanobacteriales* (methanogen) and methane production. Therefore, MiEn-LEDRAGON has the potential to be used as a feed additive rich in phytonutrients for ruminants. Nevertheless, additional in vivo trials are necessary to evaluate its effectiveness in animal production.

## Data Availability

The datasets generated and analyzed during the current study are available from the corresponding author on reasonable request.
